# Sepsis prediction in critically ill patients by platelet activation markers on ICU admission: a prospective pilot study

**DOI:** 10.1186/s40635-017-0145-2

**Published:** 2017-07-12

**Authors:** Nathalie Layios, Céline Delierneux, Alexandre Hego, Justine Huart, Christian Gosset, Christelle Lecut, Nathalie Maes, Pierre Geurts, Arnaud Joly, Patrizio Lancellotti, Adelin Albert, Pierre Damas, André Gothot, Cécile Oury

**Affiliations:** 1 Department of General Intensive Care, University Hospital of Liège, Liège, Belgium; 2 Laboratory of Thrombosis and Hemostasis, GIGA-Cardiovascular Sciences, University of Liège, Department of Cardiology, University Hospital of Liège, Liège, Belgium; 3 Laboratory of Hematology, University Hospital of Liège, Liège, Belgium; 4 Department of Biostatistics and Medico-Economic Information, University Hospital of Liège, Liège, Belgium; 5 Systems and Modeling, Department of Electrical Engineering and Computer Science, University of Liège, Liège, Belgium; 6 Gruppo Villa Maria Care and Research, Anthea Hospital, Bari, Italy

**Keywords:** Sepsis, Prediction, Flow cytometry, Platelet markers, Fibrinogen, SOFA, Biomarker

## Abstract

**Background:**

Platelets have been involved in both immune surveillance and host defense against severe infection. To date, whether platelet phenotype or other hemostasis components could be associated with predisposition to sepsis in critical illness remains unknown. The aim of this work was to identify platelet markers that could predict sepsis occurrence in critically ill injured patients.

**Methods:**

This single-center, prospective, observational, 7-month study was based on a cohort of 99 non-infected adult patients admitted to ICUs for elective cardiac surgery, trauma, acute brain injury, and post-operative prolonged ventilation and followed up during ICU stay. Clinical characteristics and severity score (SOFA) were recorded on admission. Platelet activation markers, including fibrinogen binding to platelets, platelet membrane P-selectin expression, plasma soluble CD40L, and platelet-leukocytes aggregates were assayed by flow cytometry at admission and 48 h later, and then at the time of sepsis diagnosis (Sepsis-3 criteria) and 7 days later for sepsis patients. Hospitalization data and outcomes were also recorded.

**Methods:**

Of the 99 patients, 19 developed sepsis after a median time of 5 days. These patients had a higher SOFA score at admission; levels of fibrinogen binding to platelets (platelet-Fg) and of D-dimers were also significantly increased compared to the other patients. Levels 48 h after ICU admission no longer differed between the two patient groups. Platelet-Fg % was an independent predictor of sepsis (*P* = 0.0031). By ROC curve analysis, cutoff point for Platelet-Fg (AUC = 0.75) was 50%. In patients with a SOFA cutoff of 8, the risk of sepsis reached 87% when Platelet-Fg levels were above 50%. Patients with sepsis had longer ICU and hospital stays and higher death rate.

**Conclusions:**

Platelet-bound fibrinogen levels assayed by flow cytometry within 24 h of ICU admission help identifying critically ill patients at risk of developing sepsis.

**Electronic supplementary material:**

The online version of this article (doi:10.1186/s40635-017-0145-2) contains supplementary material, which is available to authorized users.

## Background

Despite sustained research on the immune pathophysiology of sepsis, sepsis occurrence remains the leading cause of mortality (20–50%) in the intensive care unit (ICU) [1, 2]. Therefore, the identification of predictive biomarkers of sepsis is instrumental to improve ICU patients’ outcome. The Third International Consensus Task Force (Sepsis-3) defines sepsis as a “life-threatening organ dysfunction caused by a dysregulated host response to infection”. In this concept, growing experimental and preclinical evidence indicates that platelets could play an active role either in immune surveillance or in the response to infection. Indeed, in addition to their role in hemostasis and thrombosis, several studies in animal models suggest a contribution of platelets to infectious diseases due to their ability to influence innate and adaptive immune responses [3]. First, platelets may act as sentinels of the immune system. They indeed express many major receptors of the innate immune system, including most Toll-like receptors. Platelets are able to recognize molecular features of microbes and secrete many immunomodulatory mediators essential for alerting and recruiting cells of the immune system [4–7]. Second, platelets may contain infection both directly and through functional interactions with immune cells [8]. Platelets produce various antimicrobial molecules, including defensins [9], thrombocidins [10], and kinocidins, and they are able to interact with and kill bacteria directly [11]. For instance, it has been shown that activated platelets facilitate the clearance of adherent *Streptococci* in experimental infective endocarditis [12]; β-defensins released from platelets activated by the *Staphylococcus aureus* α-toxin impair bacterial growth and induce neutrophil extracellular trap formation [13]. Platelets also help trap blood pathogens on Kupffer cells in hepatic sinusoids, which limits systemic infection [14]. Notably, platelets express CD40L, an essential player in host defense against infection that mediates interactions between platelets, antigen-presenting cells, and lymphocytes [15].

In overwhelming sepsis, platelets contribute to activation of the procoagulant cascade and ensuing complications linked to microvascular thrombosis and subsequent organ dysfunction [16]. It has been demonstrated that critically ill injured adult patients, such as burn, trauma, or cardiac surgery patients, experience susceptibility to sepsis because of innate and adaptive immune reprogramming due to the insult [17, 18]. However, whether platelets may participate in dysregulated host response to infection leading to sepsis remains unclear. One recent study showed that immature platelet fractions (IPF) could predict sepsis occurrence in critically ill subjects [19]. Further, in severe trauma, platelet activation and leukocyte-platelet aggregate formation have been incriminated in the pathogenesis of tissue lesions leading to organ failure [20]. The present prospective observational study hypothesized that platelet activation markers triggered by common injuries may help predicting occurrence of sepsis in specific ICU patient populations.

## Methods

### Study patients

This was a single-center, prospective, observational, 7-month study based on a cohort of 99 consecutive adult patients, expected to stay for at least 48 h in tertiary ICU. Inclusion criteria included elective cardiac surgery (coronary artery bypass grafting or valve replacement), trauma, invasive ventilation >48 h for reasons other than sepsis, and acute brain injury (including subarachnoid, subdural, intra-parenchymal hemorrhage, and ischemic stroke). Patients were excluded from the study if they received oral or parenteral antibiotics other than for prophylaxis and if they were treated with any immunosuppressive agent except substitutive doses of corticosteroids, suffered from chronic hepatitis B or C, HIV, solid organ, or hematologic proliferative disease.

### Characteristics at ICU admission

Upon admission to ICU, the following baseline characteristics were recorded: gender, age, type of admission (surgical or medical), history of diabetes and cardiovascular disease, previous treatment by vasopressor, prophylactic antibiotics, aspirin, and anticoagulants (anti-αIIbβ3). The sequential organ failure assessment (SOFA) score was computed. Blood samples were collected within 24 h (T1) for flow cytometry analyses (see the “Flow cytometry” section below). The following laboratory parameters were also assayed: C-reactive protein (CRP, mg/ml), fibrinogen (g/l), partial thromboplastin time (PTT, s), prothrombin time index (%), platelet count (k/μl), d-dimers (μg/l), and WBC count (K/μl). The ISTH scoring system for overt disseminated intravascular coagulation (DIC) was calculated based on Toh et al. [21].

### Follow-up and sepsis occurrence

Patients were sampled again 48 h (T2) after admission, on the day of diagnosis of sepsis (Tx), and 7 days later. All blood specimens were analyzed by flow cytometry as in T1. A time line diagram is provided as Additional file 1: Figure S1. Criteria for severe sepsis or septic shock are in agreement with the new definitions of sepsis (Sepsis-3) [22]. For each study patient, the following data were also collected: length of ICU and of hospital stay (days), duration of ventilation (days) if required, administration of vasopressor during ICU admission, antibiotic treatment, use of curative antibiotics, red blood cell transfusion, plasma transfusion and platelet transfusion, and hemofiltration or intermittent hemodialysis during or after ICU stay. In case of death, time was also recorded. In case of discharge from the hospital, follow-up was at least 1 year.

### Flow cytometry

Citrated whole blood was collected through an indwelling arterial catheter. Samples were processed within maximum 1 h following blood drawing. Platelet activation levels were assessed by measuring the expression of P-selectin (PS), a marker of degranulation, and fibrinogen (Fg) binding, as a result of integrin αIIbβ3 activation, on cell surface. Specifically, blood samples were fixed and incubated with peridinin-chlorophyll protein-linked (PerCP)-anti-CD61 antibodies (BD Biosciences), fluorescein isothiocyanate-linked (FITC)-anti-fibrinogen antibodies (Dako), and phycoerythrin-linked (PE)-anti-CD62P antibodies (BD Biosciences). Levels of platelet activation markers were determined by recording medians of FITC and PE fluorescence intensity (MFI) in platelets (CD61 positive cells) and percentages (%) of fibrinogen-positive (FITC) or CD62P-positive (PE) platelets on a FACS Verse flow cytometer (BD Biosciences). Data were analyzed using the BD FACSuite software. Platelets-monocytes and platelets-neutrophils aggregates were analyzed in citrated whole blood samples using an antibody panel, including anti-CD45-V500, anti-CD14-APC (monocytes), anti-CD15-PE (neutrophils), and anti-CD61-PerCP. Medians of CD61-PerCP fluorescence intensity in CD14-positive and CD15-positive cells, and percentages of cells double positive for CD61 and CD14, or CD61 and CD15 were recorded as above. In all cases, threshold of positivity was set by use of marker-specific antibodies or their corresponding IgG isotype controls in blood samples that were left unstimulated or activated with a supra-optimal dose of collagen-related peptide. Plasma was prepared from the citrated whole blood samples to quantify plasma levels of TNFα, IL10, sCD40L, IL17A, IL6, IL7, and IFNγ, all expressed in pg/ml. Cytokine levels were measured using customized multiplex BD™ Cytometric Bead Array on the FACSVerse System. Analysis was performed with the FCAP Array™ software.

### Statistics

Results were expressed as mean and standard deviation for quantitative data and as median and interquartile range (IQR) for durations. For categorical findings, frequency tables were used. The predictive value of sepsis was assessed for each baseline variable by logistic regression analysis. Then variables significant at *P* < 0.10 were combined in a stepwise logistic regression analysis to identify independent baseline predictors of sepsis. The odds ratio (OR) with 95% confidence interval (95%CI) and ROC curve analysis with area under the curve (AUC) were used to quantify the ability of the selected predictors to discern patients who will later develop sepsis. The Youden method was applied to define an optimal cutoff point for platelet marker predictors and SOFA score. Comparisons of hospital data and outcomes between septic and non-septic patients were done by the Kruskal-Wallis test for continuous variables and the Fisher exact test for categorical variables. Data recorded on the same patients but at different time points were compared by the Wilcoxon signed rank test. Results were considered significant at the 5% critical level (*P* < 0.05). All statistical calculations were performed with SAS (version 9.4) and R (version 3.0.3).

## Results

### Baseline characteristics of patients

The baseline ICU admission characteristics of the 99 study patients are displayed in Additional file 2: Table S1. There were 60 men and 39 women aged 64 ± 15 years. The type of admission was surgical for 86 patients, and the main reason was predominantly cardiac surgery (68.7%). Seventeen patients had a history of diabetes and 79 of cardiovascular disease. Ten patients received vasopressor before admission, 67 received prophylactic antibiotics during surgery, 53 were under aspirin, 3 were under αIIbβ3 antagonist, and 14 patients were taking anticoagulant (only prophylactic doses of low molecular weight heparin). The mean SOFA score was 6.0 ± 3.3. Data of routine biological parameters and flow cytometry results upon admission and 48 h later are displayed in Additional file 1: Table S2. No difference was evidenced between aspirin (*n* = 53) or anticoagulant users (*n* = 14) and non-users in terms of their biological profile (data not shown).

### Sepsis occurrence

Of the 99 study subjects, 19 (19.2%) developed sepsis after a median time of 5 [IQR 3–7] days and 80 did not. As seen in Table 1, age, gender, type of admission, history of diabetes, use of vasopressor, anti-platelet, or anticoagulation medication use were not associated with sepsis development. By contrast, patients who later developed sepsis presented with higher SOFA score at admission. They were also predominantly admitted for acute brain surgery or prolonged ventilation and lacked prophylactic antibiotics prior to admission. Complementary results of septic compared to non-septic patients are shown in Additional file 2: Table S3.

**Table 1:** Predictive value of patient demographic and baseline clinical data for sepsis development during ICU stay

Variable	Development of sepsis^a^		*P* value^b^
	No (*N* = 80)	Yes (*N* = 19)	
Age (years)	65 ± 15	62 ± 15	0.46
Gender			0.80
Male	48 (80)	12 (20)	
Female	32 (82.1)	7 (17.9)	
Category of admission			0.70
Surgical	70 (81.4)	16 (18.6)	
Medical	10 (76.9)	3 (23.1)	
Reason for admission			0.0052
Cardiac surgery	61 (89.7)	7 (10.3)	
Acute brain injury	6 (50)	6 (50)	
Trauma	10 (76.9)	3 (23.1)	
Ventilation >48 h	3 (50)	3 (50)	
Score at admission			
SOFA	5.2 ± 2.7	9.6 ± 3.1	<0.0001
Diabetes			0.62
Yes	13 (76.5)	4 (23.5)	
No	67 (81.7)	15 (18.3)	
Cardiovascular disease			0.012
Yes	68 (86.1)	11 (13.9)	
No	12 (60)	8 (40)	
Vasopressor before the admission			0.091
Yes	6 (60)	4 (40)	
No	74 (83.2)	15 (16.8)	
Prophylactic antibiotics			0.0005
Yes	61 (91)	6 (9)	
No	19 (59.4)	13 (40.6)	
Aspirin			0.93
Yes	43 (81.1)	10 (18.9)	
No	37 (80.4)	9 (19.6)	
Anticoagulant			0.24
Yes	13 (92.9)	1 (7.1)	
No	67 (78.8)	18 (21.1)	

^a^Means ± SD for quantitative variable and numbers (%) for qualitative parameters

^b^Logistic regression

**Fig. 1:**
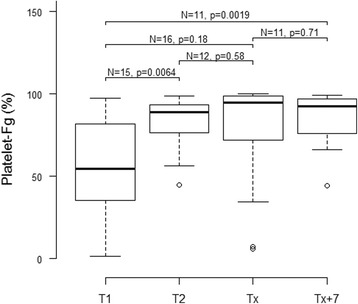
Serial measurements of levels of platelet-bound fibrinogen (platelet-Fg) for patients who developed sepsis. Platelet-Fg levels were analyzed by flow cytometry on the day of ICU admission (*T1*), after 48 h (*T2*), at the time of sepsis diagnosis (*Tx*), and 7 days later (*Tx + 7*). Median values of percentages of Fg-positive platelets and IQR are shown (*P* value: Kruskal-Wallis test)

When considering laboratory tests and flow cytometry parameters recorded within 24 h of admission to ICU (Table 2), d-dimers and fibrinogen binding to platelets (platelet-Fg expressed as MFI or %) were markedly higher (*P* < 0.001) in patients who later developed sepsis. To a lesser extent, ISTH DIC score (*P* < 0.05) also differed between septic and non-septic patients. Interestingly, levels of sCD40L, P-selectin on circulating platelets (MFI or %), platelets-monocytes, and platelets-neutrophils aggregates were not associated with sepsis occurrence. Platelet-Fg correlated weakly with platelet P-selectin (*r* = 0.32378, *P* = 0.0011, *N* = 98), and plasma levels of d-dimers (*r* = 0.35502, *P* = 0.0004, *N* = 96) and fibrinogen (*r* = 0.34592, *P* = 0.0005, *N* = 98). No significant correlation was found with platelet count (*r* = 0.071, *P* = 0.49, *N* = 98), sCD40L (*r* = −0.10377, *P* = 0.3222, *N* = 93), or cytokine levels. Flow cytometry parameters recorded 48 h after admission were not associated with sepsis occurrence, although a tendency (*P* < 0.10) remained for platelet-Fg (data not shown). When looking at serial platelet-Fg levels in patients who developed sepsis, a significant increase was observed and a peak was reached on the day of sepsis (Fig. 1). By contrast, sCD40L remained fairly stable as sepsis developed (Additional file 1: Figure S2). d-dimers and platelet P-selectin levels increased significantly from T2 to the time of sepsis diagnosis (Additional file 1: Figure S2).

**Table 2:** Predictive value of laboratory tests assessed at admission for sepsis development during ICU stay

Variable	Development of sepsis^a^		*P* value^b^
	No (*N* = 80)	Yes (*N* = 19)	
Routine			
CRP (mg/L)	14.1 ± 37.7	29.1 ± 61.7	0.053
Fibrinogen (g/L)	2.6 ± 0.99	3.3 ± 2.0	0.11
PTT (s)	14.4 ± 1.7	14.4 ± 3.3	0.78
Prothrombin Time Index (%)	66.3 ± 15.6	69.7 ± 19.4	0.61
Platelet count (10^3^/μL)	124 ± 55	133 ± 84	0.59
d-dimers (μg/L)	2617 ± 6353	4456 ± 4957	0.0032
ISTH score	1.6 ± 1.3	2.6 ± 0.9	0.041
White blood cell count (10^3^/μL)	10.0 ± 4.4	11.1 ± 5.2	0.42
Flow cytometry			
TNF-α (pg/mL)	0.17 ± 0.8	0.65 ± 1.5	0.091
IL-10 (pg/mL)	19.4 ± 90	9.4 ± 16.2	0.50
sCD40L (pg/mL)	88.8 ± 81.8	53.3 ± 43.6	0.89
IL-17A (pg/mL)	9.1 ± 12.2	7.6 ± 15.5	0.31
IL-6 (pg/mL)	459 ± 2673	162 ± 164	0.94
IL-7 (pg/mL)	2.9 ± 3.7	1.4 ± 1.7	0.65
IFN-γ (pg/mL)	0.14 ± 0.91	0	0.99
Platelet-Fg (%)	28.1 ± 27.8	56.5 ± 31.2	0.0054
Platelet-Fg (MFI)	1770 ± 1266	2752 ± 1359	0.0026
Platelet-PS (%)	2.9 ± 2.4	3.5 ± 2.7	0.61
Platelet-PS (MFI)	29.8 ± 15.1	37 ± 18.3	0.068
Platelets-neutrophils (%)	3.4 ± 5	4.4 ± 6.2	0.72
Platelets-neutrophils (CD61 MFI)	313 ± 127	323 ± 137	0.72
Platelets-monocytes (%)	19.8 ± 23.1	22.1 ± 25.5	0.73
Platelets-monocytes (CD61 MFI)	1413 ± 2462	1569 ± 3182	0.82

Null values for TNF-α and IFN-γ correspond to values under the level of detection (3.8 pg/ml)

*Platelet-Fg* platelet-bound fibrinogen, *platelet-PS*, platelets expressing P-selectin on their surface, *MFI* median fluorescence intensity, *%* percentage of positive cells for the indicated marker

^a^Results are expressed as means ± SD

^b^Logistic regression

### Platelet markers at admission and sepsis prediction

**Fig. 2:**
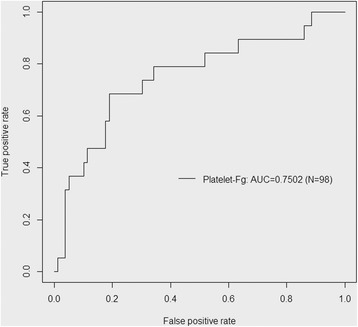
Predictive value of platelet-Fg (%) obtained at admission. ROC curve analysis of sepsis prediction based on platelet-Fg is shown

All potential predictors of sepsis (*P* < 0.10) recorded at ICU admission (T1) were combined into a stepwise logistic regression analysis. As diagnosis of sepsis includes organ dysfunction, SOFA score was not included in our regression model. It turned out that platelet-Fg % levels at T1 (*P* = 0.0031) and admission for acute brain injury (*P* = 0.012) were the only independent predictors of sepsis occurrence. By ROC curve analysis (Fig. 2), an optimal cutoff point equal to 50% was derived for platelet-Fg % (AUC = 0.75) to discern patients who will later develop sepsis from those who will not. The number of patients who developed sepsis was respectively equal to 13 (46.4%) for the 28 patients with platelet-Fg >50% and to 6 (8.6%) for the 70 patients with platelet-Fg <50% (data missing for one patient). As shown in Table 3, when accounted for SOFA score at admission (cutoff value of 8), in patients with elevated SOFA and platelet-Fg >50%, the risk of sepsis rose up to 85.7%. By contrast, in patients with low SOFA and platelet-Fg <50%, the occurrence of sepsis was almost negligible (3.8%).

**Table 3:** Risk stratification of patients according to sepsis development during ICU stay

	SOFA <8		SOFA ≥8	
	Platelet-Fg (%)		Platelet-Fg (%)	
Development of sepsis	<50	≥50	<50	≥50
Yes (*N* = 19)	2	1	4	12
No (*N* = 79)	51	13	13	2
Total	53	14	17	14
Risk of sepsis (%)	3.8	7.1	23.5	85.7

SOFA score and Platelet-Fg (%) plasma levels recorded on admission

## Discussion

The major findings of this study concern the clear relationship between patient levels of fibrinogen binding to circulating platelets (platelet-Fg) measured upon ICU admission and sepsis occurrence, regardless of the patient’s baseline clinical characteristics. In particular, the study demonstrated that for patients presenting a SOFA score ≥8, platelet-Fg % level above 50 predicted sepsis with a high accuracy. Importantly, neither platelet membrane-bound P-selectin expression plasma levels of sCD40L nor any other standard hemostasis parameter showed similar predictive value as platelet-Fg. The optimal timing of measurement was also determined since only levels obtained within 24 h after ICU admission and not 48 h later were associated with sepsis occurrence, thus saving blood sampling in future studies. Platelet-Fg levels can be obtained in 1 h by using whole blood flow cytometry in unstimulated samples. Thus, this work provides the clinician with a simple and practical tool to assess the risk of sepsis in critically ill patients admitted to the ICU.

To date, several clinical studies investigated platelet markers in various conditions of critical illness. However, none of them searched for a potential association of these platelet markers with a risk for sepsis. Most of these studies described altered platelet phenotype in injured patients, characterized by either differential expression of platelet activation markers or platelet dysfunction as compared to healthy controls [23–28]. In ischemic stroke, two studies showed increased expression of platelet P-selectin and fibrinogen binding to platelets as compared to controls [25, 29]. The latter finding is interesting in view of our results, in particular since predisposition to severe pneumonia is clinically well established in such patients [30, 31]. Unfortunately, no association was searched between high levels of the biomarker and pneumonia. Several other clinical studies focusing on platelets as potential biomarkers for sepsis diagnosis and prognostication have been carried out but almost all concerned patients with sepsis as an inclusion criterion [32, 33].

Despite multiple experimental data demonstrating antimicrobial activity of platelets and a role for platelet aggregation in limiting pathogen growth and dissemination in the vasculature [3, 6], direct clinical evidence from human studies was lacking and there are no epidemiologic data showing that platelet function inhibition affects sepsis prediction or prognosis. The present observational prospective study provides the first clinical evidence that, in patients with critical illness and related organ dysfunction, platelets may intervene in the dysregulated host response to infection leading to sepsis. Although demonstration of a causal link requires further investigation, we speculate that injury-associated platelet activation and subsequent fibrinogen binding may alter platelet ability to recognize bacterial components, some of which are ligands of αIIbβ3 [34, 35], and affect their ability to alert and recruit cells of the immune system [8]. Our observation that platelet-Fg weakly correlates with d-dimer levels suggests that fibrinogen binding to platelets and the activation of coagulation could be driven by the same factors. In injured patients, plasma fibrinogen would both bind platelets and be actively converted into fibrin; fibrinolysis would then increase d-dimer levels.

Antiplatelet drugs have beneficial and detrimental effects in systemic inflammation and in organ dysfunction, as shown in preclinical models and in humans [36–38]. Their usage has been variably associated with sepsis prognosis [39, 40]. In this study, we found no protective effect of aspirin against sepsis [41]. Our results are in line with a recent propensity-based analysis of 972 patients admitted for sepsis in which no association between aspirin therapy and sepsis prognosis could be evidenced [42]. Our results however differ in that they encompassed the period before sepsis, a period during which the abovementioned authors could not assess the potential benefits of aspirin. In addition, we could not find any association between aspirin therapy and the levels of platelet activation, which suggests that platelet activation pathways independent of thromboxane A_2_ production could be involved in the patient’s platelet response to injury.

### Limitations

The study has a number of limitations. The small sample size and the predominance of elective surgical patients call for caution when interpreting results. Also, possible confounders such as immunomodulatory properties of anesthetic drugs were not taken into account at this stage. The findings of this pilot study call for a confirmatory prospective evaluation focusing on fibrinogen levels on platelets in a larger cohort. In our study, the platelet activation markers analyzed, namely levels of fibrinogen, platelet P-selectin expression, platelets-leukocytes aggregates, and sCD40L, behaved differently in their ability to predict sepsis development, which might reflect differences in platelet activation mechanisms or sequences. It has indeed been proposed that platelet activation, in terms of P-selectin expression and fibrinogen binding, and release of immunological molecules (sCD40L, RANTES) result from independent signaling pathways [43]. The utility of other markers, such as platelet microparticles or soluble glycoprotein VI, should be analyzed, since the latter is shed from platelet surface and increases in patients with DIC [44].

## Conclusions

In critically ill patients with comorbidities and post-trauma or post-surgical injury, platelet abnormalities are associated with altered host defense mechanisms. We actually found that admission levels of fibrinogen binding to platelets of ICU patients were predictive of later sepsis acquisition. Combining it with stratification based on SOFA score at admission has a higher predictive ability. Hence, our observations could trigger non-specific preventive interventions such as better supportive care or prophylactic antibiotics as well as research aiming at developing a specific therapeutic tool. Also, the fact that the identified marker was independent of aspirin use might have important future therapeutic implications regarding its actual worldwide implementation of primary or secondary prophylaxis.

## Additional files

Additional file 1: Figure S1. Follow-up and sepsis occurrence. Timeline of samplings. **Figure S2.** Serial measurements of platelet markers and d-dimers for patients who developed sepsis. (DOCX 49 kb)

Additional file 2: Table S1. Baseline clinical characteristics of study patients (*n*=99). **Table S2.** Baseline and 48-h biological characteristics of study patients (*n*=99). **Table S3.** Comparison of ICU- and hospital-related characteristics of patients with and without sepsis. (PDF 157 kb)

## Abbreviations

AUC: Area under curve
CI: Confidence interval
CRP: C-reactive protein
Fg: Fibrinogen
MFI: Median of fluorescence intensity
OR: Odds ratio
PS: P-selectin
ROC: Receiver operating characteristic
SOFA: Sequential organ failure assessment
