# Supplementary material for: Sepsis prediction in critically ill patients by platelet activation markers on ICU admission: a prospective pilot study
Source: Intensive Care Med Exp. 2017 Jul 12;5:32. doi: 10.1186/s40635-017-0145-2 (PMC5505890; doi:10.1186/s40635-017-0145-2)
Supplement: Supplementary file 1 — Follow-up and sepsis occurrence. Timeline of samplings. Figure S2. Serial measurements of platelet markers and d-dimers for patients who developed sepsis. (DOCX 49 kb) [file 40635_2017_145_MOESM1_ESM.pdf]

Sepsis prediction in critically ill patients by platelet activation markers on ICU admission: a prospective pilot study

*Nathalie Layios[1,2]; *Céline Delierneux[2]; Alexandre Hego[2]; Justine Huart[2]; Christian Gosset[3]; Christelle Lecut[3]; Nathalie Maes[4]; Pierre Geurts[5]; Arnaud Joly[5]; Patrizio Lancellotti[2,6]; Adelin Albert[4]; Pierre Damas[1]; André Gothot[3]; Cécile Oury[2]

Additional file 2

Figures

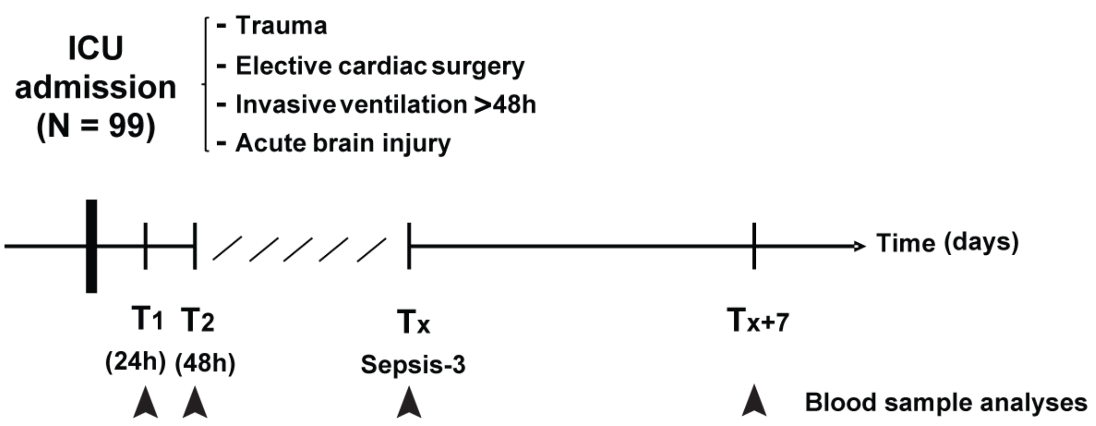

Additional Figure 1. Follow-up and sepsis occurrence. Timeline of samplings.

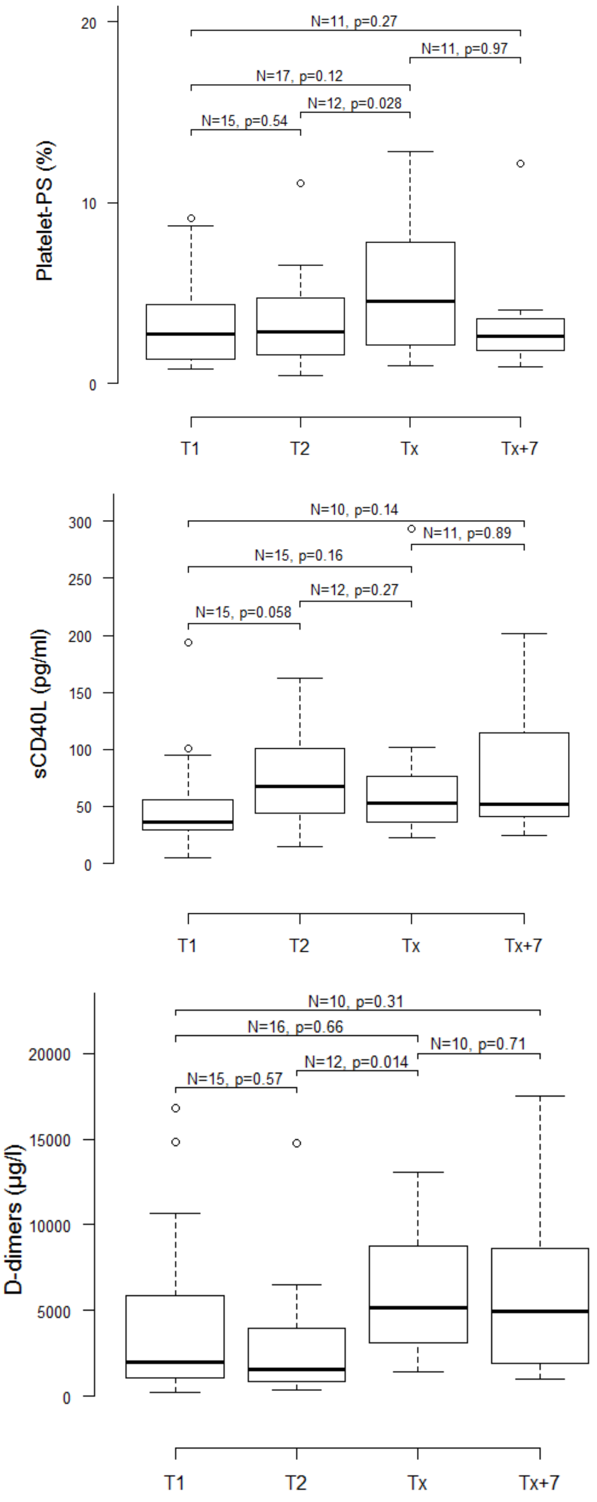

Additional Figure 2. Serial measurements of platelet markers and D-dimers for patients who developed sepsis. Percentages of platelets exposing P-selectin (Platelet-PS), levels of plasma sCD40L, and D-dimers were analyzed on day of ICU admission (T1), after 48h (T2), at the time of sepsis diagnosis (Tx) and 7 days later (Tx+7). Median values and IQR are shown (P-value: Kruskal-Wallis test).
