# Supplementary material for: Sepsis prediction in critically ill patients by platelet activation markers on ICU admission: a prospective pilot study
Source: Intensive Care Med Exp. 2017 Jul 12;5:32. doi: 10.1186/s40635-017-0145-2 (PMC5505890; doi:10.1186/s40635-017-0145-2)
Supplement: Supplementary file 2 — Baseline clinical characteristics of study patients (n=99). Table S2. Baseline and 48-h biological characteristics of study patients (n=99). Table S3. Comparison of ICU- and hospital-related characteristics of patients with and without sepsis. (PDF 157 kb) [file 40635_2017_145_MOESM2_ESM.docx]

**Sepsis prediction in critically ill patients by platelet activation markers on ICU admission: a prospective pilot study**

***Nathalie Layios^1,2^; *Céline Delierneux^2^; Alexandre Hego^2^; Justine Huart^2^; Christian Gosset^3^; Christelle Lecut^3^; Nathalie Maes**^4^; Pierre Geurts^5^; Arnaud Joly^5^; **Patrizio Lancellotti^2,6^; Adelin Albert**^4^; **Pierre Damas^1^; André Gothot^3^; Cécile Oury^2^**

**Additional file 1**

**Tables 1-3**

Additional Table 1 - Baseline clinical characteristics of study patients (n=99)

| Variable | Baseline^1^ |
| --- | --- |
| Age (years) | 64 ± 15 |
| Gender (male) | 60 (60.9) |
| Category of admission |  |
| Surgical | 86 (86.9) |
| Medical | 13 (13.1) |
| Reason for admission |  |
| Cardiac surgery | 68 (68.7) |
| Acute brain injury | 12 (12.1) |
| Trauma | 13 (13.1) |
| Ventilation >48h | 6 (6.1) |
| Score at admission |  |
| SOFA | 6.0 ± 3.3 |
| Diabetes | 17 (17.2) |
| Cardiovascular disease | 79 (79.8) |
| Vasopressor before the admission | 10 (10.1) |
| Prophylactic antibiotics | 67 (67.7) |
| Aspirin | 53 (53.5) |
| Anticoagulant | 14 (14.1) |

^1^Mean ± SD for quantitative variable and number (%) for qualitative parameters

Additional Table 2 - Baseline and 48-hour biological characteristics of study patients (n=99)

| Variable | Baseline | 48h |
| --- | --- | --- |
| Routine |  |  |
| CRP (mg/L) | 17.1 ± 43.4 | 93.9 ± 182.6 |
| Fibrinogen (g/L) | 2.7 ± 1.3 | NA |
| PTT (s) | 14.4 ± 2.1 | NA |
| Prothrombin Time Index (%) | 66 ± 16.4 | NA |
| Platelet count (10^3^/μL) | 126 ± 61 | 111 ± 58 |
| D-dimers (μg/L) | 2977 ± 6124 | 2100 ± 4111 |
| DIC score | 1.8 ± 1.3 | NA |
| White blood cell count (10^3^/μL) | 10.2 ± 4.6 | 11.3 ± 3.5 |
| Flow cytometry |  |  |
| TNF-α (pg/mL) | 0.27 ± 1.0 | 0.17 ± 0.82 |
| IL-10 (pg/mL) | 17.4 ± 81.2 | 2.8 ± 7.2 |
| sCD40L (pg/mL) | 82 ± 77.1 | 89.9 ± 64.1 |
| IL-17A (pg/mL) | 8.9 ± 12.2 | 7.8 ± 11 |
| IL-6 (pg/mL) | 402 ± 2404 | 122 ± 266 |
| IL-7 (pg/mL) | 2.6 ± 3.5 | 2.7 ± 4.4 |
| IFN-γ (pg/mL) | 0.12 ± 0.83 | 0.04 ± 0.21 |
| Platelet-Fg (%) | 33.6 ± 30.5 | 70.2 ± 25.5 |
| Platelet-Fg (MFI) | 1960 ± 1335 | 3388 ± 1301 |
| Platelet-PS (%) | 3 ± 2.4 | 3.1 ± 2.0 |
| Platelet-PS (MFI) | 33.6 ± 30.5 | 70.2 ± 25.5 |
| Platelets-neutrophils (%) | 3.6 ± 5.2 | 3.3 ± 2.9 |
| Platelets-neutrophils (CD61 MFI) | 315 ± 128 | 302 ± 82 |
| Platelets-monocytes (%) | 20.3 ± 23.4 | 20.0 ± 16.5 |
| Platelets-monocytes (CD61 MFI) | 1443 ± 2597 | 1102 ± 1149 |

Results are expressed as means ± SD. Platelet-Fg, platelet-bound fibrinogen; platelet-PS, platelets expressing P-selectin

on their surface; null values for TNF-α and IFN-γ correspond to values under the level of detection (3.8pg/ml); MFI,

Median fluorescence intensity; %, percentage of positive cells for the indicated marker; NA, not available

Additional Table 3 – Comparison of ICU- and hospital-related characteristics of patients with and without sepsis

| Variable | Patients with sepsis^1^ | | P-value^2^ |
| --- | --- | --- | --- |
|  | No | Yes |  |
| Duration of ICU stay (days) | 3 (2-4) | 15 (10-22) | <0.0001 |
| Duration of hospital stay (days) | 11 (9-16) | 26 (16-71) | <0.0001 |
| Ventilation | 12 (15.0) | 16 (88.9) | <0.0001 |
| Duration of ventilation (days) | 1 (1-1) | 10 (6-15) | <0.0001 |
| Vasopressor during the admission in ICU | 14 (17.5) | 12 (66.7) | <0.0001 |
| Antibiotic treatment | 62 (77.5) | 12 (66.7) | 0.24 |
| Curative antibiotics | 1 (1.3) | 6 (33.3) | 0.0001 |
| Red blood cell transfusion | 17 (21.3) | 6 (33.3) | 0.37 |
| Plasma transfusion | 9 (11.3) | 2 (11.1) | 0.99 |
| Platelet transfusion | 7 (8.9) | 2 (11.1) | 0.68 |
| Hemofiltration or intermittent haemodialysis | 0 (0) | 6 (33.3) | <0.0001 |
| 28-day mortality | 6 (7.5) | 6 (33.3) | 0.0055 |
| 90-day mortality | 7 (8.9) | 7 (38.9) | 0.0026 |

^1^Medians and IQR for duration values and numbers (%) for qualitative parameters

^2^P-value of Kruskal-Wallis test or Fisher exact test
